# Comparative studies of Al-doped ZnO and Ga-doped ZnO transparent conducting oxide thin films

**DOI:** 10.1186/1556-276X-7-639

**Published:** 2012-11-22

**Authors:** Min-Chul Jun, Sang-Uk Park, Jung-Hyuk Koh

**Affiliations:** 1 Department of Electronic Materials Engineering, Kwangwoon University, Seoul, 139-701, South Korea

**Keywords:** AZO, GZO, Transparent conducting oxide, Thin films, Spin-coating

## Abstract

We have investigated the influences of aluminum and gallium dopants (0 to 2.0 mol%) on zinc oxide (ZnO) thin films regarding crystallization and electrical and optical properties for application in transparent conducting oxide devices. Al- and Ga-doped ZnO thin films were deposited on glass substrates (corning 1737) by sol–gel spin-coating process. As a starting material, AlCl_3_⋅6H_2_O, Ga(NO_3_)_2_, and Zn(CH_3_COO)_2_⋅2H_2_O were used. A lowest sheet resistance of 3.3 × 10^3^ Ω/□ was obtained for the GZO thin film doped with 1.5 mol% of Ga after post-annealing at 650°C for 60 min in air. All the films showed more than 85% transparency in the visible region. We have studied the structural and microstructural properties as a function of Al and Ga concentrations through X-ray diffraction and scanning electron microscopy analysis. In addition, the optical bandgap and photoluminescence were estimated.

## Background

Transparent conducting oxide (TCO) films have been intensively investigated for optical and electrical applications, such as flat-panel displays, liquid crystal displays, organic light-emitting diodes, thin-film transistors, and thin-film solar cells [1-4]. TCO thin films should have low resistivity, high transmittance in the visible region (400 to 800 nm), and high thermal/chemical stability [5,6]. In most cases, indium tin oxide (ITO) has been widely employed as a TCO material because of its superb electrical and optical properties. However, ITO has low stability, high toxicity, and high cost and is a rare material, motivating efforts to develop alternatives [7].

Recently, zinc oxide (ZnO) has been regarded as a promising candidate to replace ITO due to its low cost and excellent properties as compared with ITO. For the purpose of improving the electrical conductivity and optical transmittance of ZnO thin films, group III elements such as boron, aluminum, gallium, and indium are usually introduced to ZnO [8]. Undoped and doped ZnO thin films have been prepared by a variety of thin film deposition techniques, such as chemical vapor deposition, DC and RF magnetron sputtering, electron beam evaporation, thermal plasma, pulsed laser deposition, metal organic chemical vapor deposition, spray pyrolysis, and sol–gel method [9-17].

In this study, Al-doped ZnO (hereafter AZO) and Ga-doped ZnO (hereafter GZO) thin films were prepared by sol–gel spin-coating method since this particular technique offers several advantages, such as large deposition area, simple equipment, low fabrication cost, and high homogeneity of the precursor. We compare the effects of Al and Ga dopants on the microstructure, electrical, and optical properties of the AZO and GZO thin films as a function of doping concentration.

## Experimental details

Thin films were prepared by sol–gel spin-coating method. As starting materials, Ga(NO_3_)_2_, AlCl_3_⋅6H_2_O, and Zn(CH_3_COO)_2_⋅2H_2_O were used. As solvent and stabilizer, 2-methoxyethanol and monoethanolamine (MEA) were used, respectively. Zinc acetate dihydrate was first dissolved in a mixture of 2-methoxyethanol and MEA solution at room temperature. The molar ratio of MEA to zinc acetate dihydrate was maintained at 1.0, and the concentration of zinc acetate dihydrate was 0.7 mol/L. In order to study the influence of Al and Ga dopant concentrations on the properties of Al-doped and Ga-doped ZnO thin films, the concentrations were varied at 0, 0.5, 1.0, 1.5, and 2.0 mol% with respect to Zn. The solutions were stirred at 60°C for 2 h to yield a clear and homogeneous solution. Thereafter, Corning 1737 glass (Corning Inc., Corning, NY, USA) was ultrasonically cleaned in acetone, methanol and DI water for 5 min, respectively. AZO and GZO films were then deposited on glass substrates (Corning 1737) by sol–gel spin-coating method. Spin coating was performed at room temperature, with a rate of 3,000 rpm for 20 s. After being deposited by sol–gel spin coating, the films were preheated at 300°C for 10 min on a hot plate to evaporate the solvent and remove organic residuals. The procedures from coating to drying were repeated six times. The films were then placed in a furnace and post-heated in air at 650°C for 1.5 h.

The crystalline structures of the specimens were analyzed by X-ray diffraction (XRD) patterns. XRD 2*θ* scans were carried out by employing a Rigaku X-ray diffractometer (Rigaku Corporation, Tokyo, Japan) with a Cu-K*α* source (*λ* = 0.154056 nm). The surface microstructure was observed by SEM (Hitachi S-4300, Hitachi High-Tech, Minato-ku, Tokyo, Japan). Electrical resistance was measured using four-point probe method and Hall measurement system. Optical transmittance measurements were carried out using a UV–vis spectrophotometer. Photoluminescence (PL) spectra were recorded using a PL spectrometer excited with a 325-nm He-Cd laser at room temperature.

## Discussion

**Figure 1:**
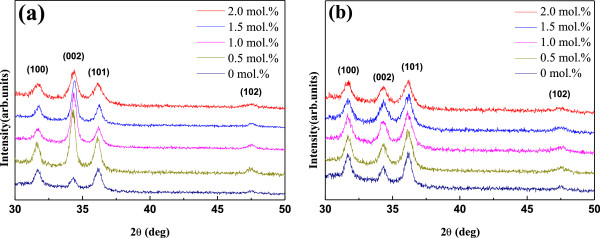
**XRD scans with Cu-K*α *radiation for thin films at different Al and Ga dopant concentrations.** (**a**) AZO and (**b**) GZO thin films.

The XRD patterns of AZO and GZO thin films at different Al and Ga doping concentration annealed at 650°C in air for 1.5 h are shown Figure 1a,b, respectively. All of the undoped, Al-, and Ga-doped ZnO thin films were polycrystalline and have a hexagonal wurtzite crystal structure [18]. As shown in Figure 1a, the AZO thin films show a preferentially *c*-axis orientation normal to the substrate surface after adding the Al dopant, and the intensity of the (002) plane decreased with increasing Al doping concentration from 0.5 to 2.0 mol%. This indicates that excessive Al doping deteriorates the crystallinity of the films, which may be due to the formation of stress by the smaller radius of Al^3+^ ions (0.054 nm) compared with Zn^2+^ ions (0.074 nm) [19]. Unlike the Al dopant, the Ga dopant has no significant influence on the crystal structure of ZnO, as shown in Figure 1b. A possible reason could be the smaller difference in radius between Ga^3+^ ions (0.062 nm) and Zn^2+^ ions than between Al^3+^ ions and Zn^2+^ ions. Thus, Ga^3+^ ions minimize the influence on ZnO crystallinity when doped into ZnO films [20,21].

To confirm the relationship between crystallinity and Al and Ga dopants, we investigated the stress in the direction of the *c*-axis. The strain (*ε*) along the *c*-axis in the AZO and GZO thin films can be defined as *ε* = (*c*_film_ − *c*_bulk_) / *c*_bulk_, where *c*_bulk_ (5.200 Ǻ ) is the unstrained lattice parameter (American Society for Testing and Materials) and *c*_film_ is measured by XRD. The lattice constant *c* can be calculated by the following formula [22]:

(1) $$dhkl^{2}={\left(\frac{4\left(h^{2}+k^{2}+hk\right)}{3a^{2}}+\frac{l^{2}}{c^{2}}\right)}^{-1}\text{.}$$

**Figure 2:**
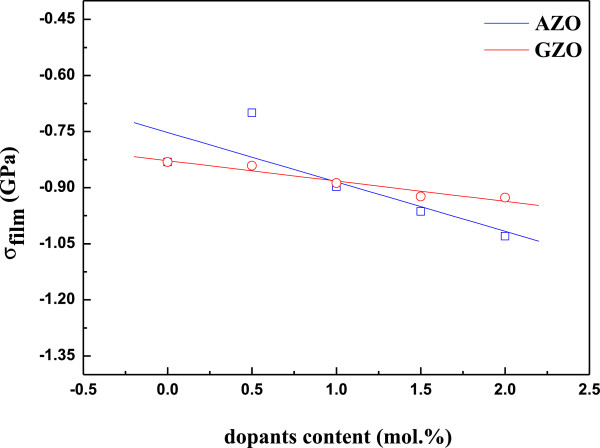
Residual stress of AZO and GZO thin films at different Al and Ga dopant concentrations.

Based on the biaxial strain model, the stress (*σ*) in the film can be calculated by the following formula, which is valid for a hexagonal lattice:

(2) $$\sigma =\frac{2{c^{2}}_{13}-c_{33}\left(c_{11}+c_{12}\right)}{2c_{13}}\times \frac{c_{\text{film}}-c_{\text{bulk}}}{c_{\text{bulk}}}\text{,}$$

where *a* and *c* are the lattice constants, and *d*_*hkl*_ is the crystalline plane distance for indices *h*, *k*, and *l*. According to Equation 1, the lattice constant *c* is equal to 2*d*_*hkl*_ for the (002) diffraction peak. The elastic constants *c*_*ij*_ of single-crystalline ZnO of *c*_11_ = 208.8, *c*_12_ = 119.7, *c*_13_ = 104.2, and *c*_33_ = 213.8 have been used [23]. Equation 2 can be simplified to *σ*_film_ = −233 × *ε* (GPa); the negative sign indicates compressive stress. Figure 2 shows that the calculated stress in the direction of the *c*-axis of AZO and GZO thin films was fitted by the power law, and the results showed a tendency of increase in the calculated stress with increasing dopant concentrations. By adding Al dopant to ZnO, the calculated stress in the direction of the *c*-axis was decreased, and an increase of Al dopant from 0.5 to 2.0 mol% resulted in the calculated stress increasing again, while there was little change in GZO. This is an expected result due to the bigger difference in radius between Al^3+^ ions and Zn^2+^ ions than with Ga^3+^ ions and Zn^2+^ ions.

**Figure 3:**
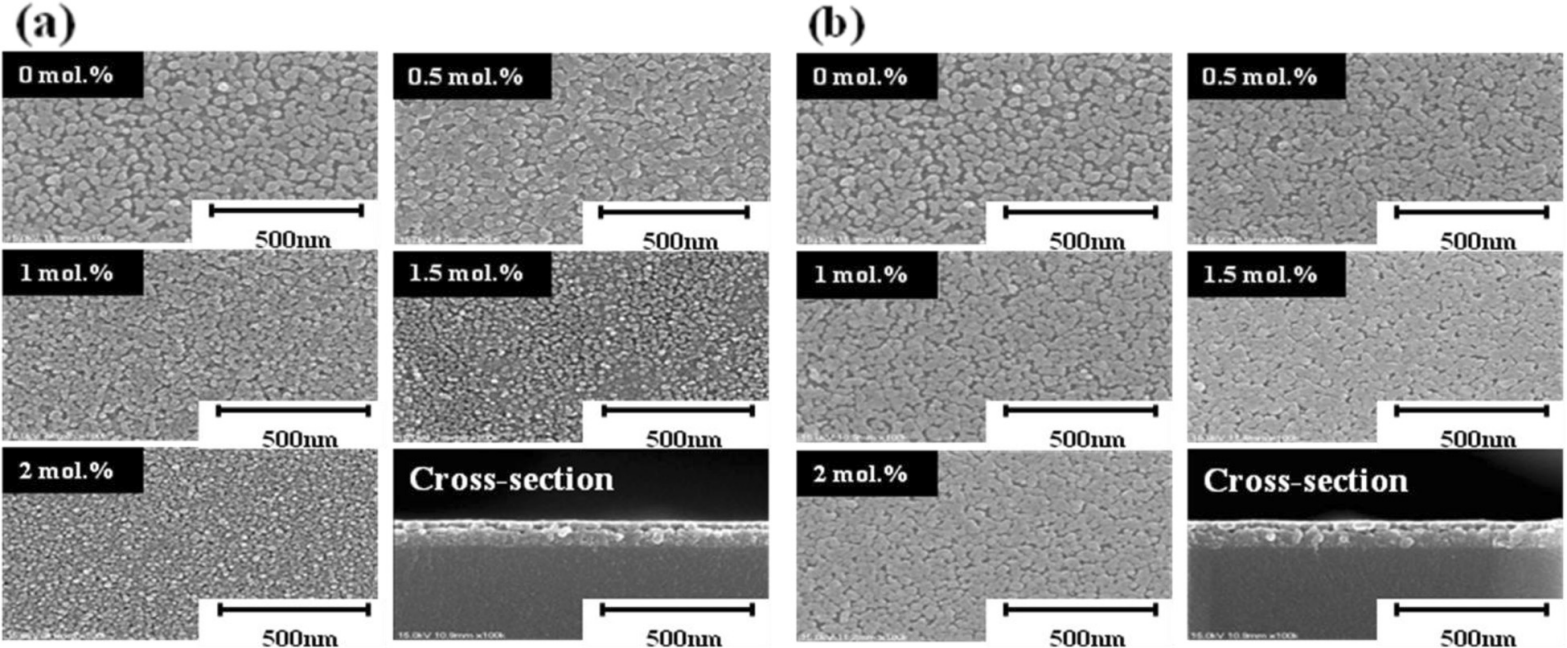
**SEM micrographs of thin films at different Al and Ga dopant concentrations.** (**a**) AZO and (**b**) GZO thin films.

As the microstructure of AZO and GZO thin films have an influence on the electrical and optical properties for optoelectronic devices, it is very important to investigate the surface morphology of AZO and GZO thin films. Figure 3 shows the surface and cross section of the AZO (Figure 3a) and GZO (Figure 3b) thin films at different Al and Ga doping concentrations (0, 0.5, 1.0, 1.5, and 2.0 mol%). The microstructures of AZO and GZO thin films are homogeneous, and the thickness of the final film is approximately 150 nm. Also, the density of microstructure was increased with increasing dopant content. We attribute that by increasing the dopant content, the grain size of thin films was decreased and then small grains make no pores. In the case of Al doping, by increasing the Al doping concentration, the grain size obtained gradually decreased, which is considered in light of two possible reasons: (1) the increasing number of nucleation leading to the formation of small grains during incorporation of the dopant into the host material and (2) the disturbance of grain growth by stress due to the difference in ion radius between zinc and aluminum [24]. However, when viewing the case of GZO, the second reason seems more suited since the grain size of the GZO thin films scarcely changed with increasing Ga concentration.

**Figure 4:**
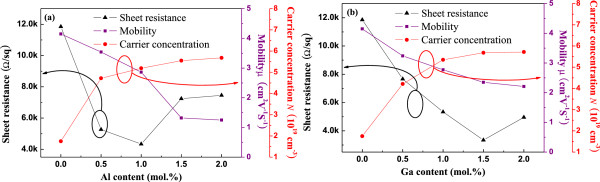
**Thin films' sheet resistance, Hall mobility, and carrier concentration at different Al and Ga dopant concentrations.** (**a**) AZO and (**b**) GZO thin films.

Figure 4 shows the sheet resistance, Hall mobility, and carrier concentration of AZO and GZO thin films at different Al and Ga doping concentrations. The sheet resistance decreased with increasing Al or Ga doping concentration. The lowest sheet resistances were 4.3 × 10^3^ and 3.3 × 10^3^ Ω/□ for 1.0-mol% Al and 1.5-mol% Ga, respectively. These decreased sheet resistance values might have been results of the increase in carrier concentration. The increase in carrier concentration of AZO and GZO thin films was due to the substitutional incorporation of Al^3+^ and Ga^3+^ ions at Zn^2+^ cation sites or the incorporation of Al or Ga ions in interstitial positions. However, with increase in the Al and Ga doping concentrations above 1.0 and 1.5 mol%, the sheet resistance started to increase. We attribute this increased sheet resistance to the decreased mobility of carriers caused by high carrier concentration, and thus, the decrease in mobility of carriers may have been due to ionized impurity scattering [25]. It is also found that the carrier mobility of AZO thin films was lower than that of GZO thin films because of the difference in the grain size between the films, as shown in Figure 3. The decrease in grain size produces more grain boundary scattering.

**Figure 5:**
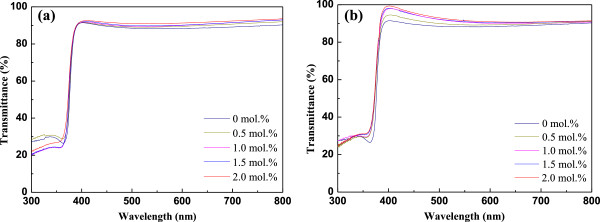
**Optical transmittance spectra of thin films at different Al and Ga dopant concentrations.** (**a**) AZO and (**b**) GZO thin films.

Optical transmittance spectra of AZO and GZO thin films at different Al and Ga doping concentrations are compared in Figure 5, in the wavelength range of 300 to 800 nm. All films exhibited a transmittance higher than 85% within the visible region, with a sharp fundamental absorption edge. In particular, the absorption edge is blueshifted with increasing Al or Ga doping concentration, which indicates broadening of the optical bandgap. Typically, the blueshift of the absorption edge of the AZO and GZO films is associated with an increase of the carrier concentration blocking the lowest states in the conduction band, which is well known as the Burstein-Moss effect [26,27].

**Figure 6:**
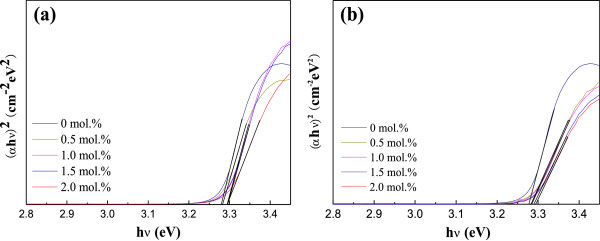
**Plot of (αhν)**^**2 **^**versus hν for thin films at different Al and Ga dopant concentrations.** (**a**) AZO and (**b**) GZO thin films.

From Figure 5, it was also found that absorption edges shift toward lower wavelength value by increasing Al or Ga doping concentration. This shift was confirmed by representing the absorbance squared versus hν in Figure 6. The absorption coefficient data were used to determine the optical bandgap, *E*_g_, using the following relation [28]:

(3) $$\alpha hv\approx {\left(hv-E_{\text{g}}\right)}^{1/2}\text{,}$$

where h*ν* is the photon energy. The absorption coefficient *α* was obtained from the transmittance data using the relation *α* = (1/*d*)ln(1/*T*), where *d* and *T* are the thickness and the transmittance of the films, respectively. Accordingly, the optical bandgap can be obtained by extrapolating the corresponding straight lines downwards to the photon energy axis in the Tauc plot [29]. The optical bandgap increased in accordance with an increase in the Al and Ga doping concentrations. The value of AZO thin films is about from 3.28 to 3.29 eV and for GZO thin films is from approximately 3.28 to 3.295 eV. According to the Burstein-Moss effect, the broadening of the optical bandgap is given as follows:

(4) $$\Delta E_{g}=\left(\frac{h^{2}}{2m_{\text{vc}}^{*}}\right)\;{\left(3\pi^{2}n\right)}^{2/3}\text{,}$$

where ∆*E*_g_ is the shift of the doped semiconductor compared to undoped semiconductor, *m*_vc_^∗^ is the reduced effective mass, *h* is Plank's constant, and *n* is the carrier concentration. According to this equation, the optical bandgap would increase with increasing carrier concentration.

**Figure 7:**
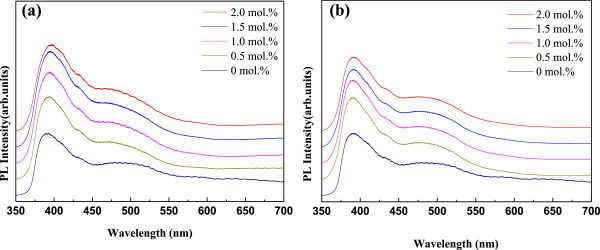
**The PL spectra of thin films at different Al and Ga dopant concentrations.** (**a**) AZO and (**b**) GZO thin films.

Figure 7 shows the PL spectra obtained at room temperature for AZO and GZO thin films with respect to Al and Ga concentrations, and the results are found to be dependent on these concentrations. Near-band-edge (NBE) emissions at about 390 nm and weak deep-level (DL) emissions are observed in all films. The strong NBE emissions originated from the free exciton recombination, and the DL emissions are associated with oxygen defects [30,31]. The green emission resulted from the recombination of electrons with holes trapped in singly ionized oxygen vacancies, which are commonly made in oxygen-deficient conditions. It is also observed that with the increase of Al or Ga concentration, the orange emission peaks disappear. This is because Al or Ga ions exist as Al^3+^ and Ga^3+^ and Zn ions as Zn^2+^. When Al or Ga are doped in ZnO, Al and Ga ions can consume residual O ions and decrease the concentration of interstitial oxygen in the AZO thin films.

## Conclusion

Aluminum- or gallium-doped ZnO thin films were prepared by sol–gel spin-coating method for TCO applications. All films had a hexagonal wurtzite crystal structure, and a minimum sheet resistance of 3.3 × 10^3^ Ω/□ was obtained for 1.5-mol% Ga-doped ZnO thin film. We also found that Al and Ga dopants acted as electrical dopants at the initial doping concentration but as impurities at greater doping concentration. The transmittance of the AZO and GZO thin films was higher than 85% in the visible region, and the optical bandgap of the AZO and GZO thin films became broader with increasing Al or Ga dopant concentration because of the Burstein-Moss effect. In conclusion, the structural, morphological, electrical, and optical characteristics of AZO and GZO thin films were observed, and Ga doping seems to be more effective than Al doping.

## Competing interests

The authors declare that they have no competing interests.

## Authors’ contributions

MCJ and SUP carried out the experiments and measurements. MCJ and JHK performed the analysis and prepared the manuscript. JHK had been guiding the research. All authors read and approved the final manuscript.

## References

[B1] Ohyama M Kozuka H Yoko T Sol–gel preparation of transparent and conductive aluminum-doped zinc oxide films with highly preferential crystal orientation J Am Ceram Soc 1998 81 1622

[B2] Cheong KY Muti N Ramanan SR Electrical and optical studies of ZnO:Ga thin films fabricated via the sol–gel technique Thin Solid Films 2002 410 142 10.1016/S0040-6090(02)00286-9

[B3] Lan JH Kanicki J Catalano A Keane J Boer WD Gu TJ Patterning of transparent conducting oxide thin films by wet etching for a-Si:H TFT-LCDs Electron Mater 1996 25 1806 10.1007/BF02657158

[B4] Rech B Wagner H Potential of amorphous silicon for solar cells. Appl. Phys. A Mater Sci Process 1999 69 155 10.1007/s003390050986

[B5] Gordillo G Calderon C Properties of ZnO thin films prepared by reactive evaporation Solar Energy Mater. Solar Cells 2001 69 251 10.1016/S0927-0248(00)00394-9

[B6] Oztas M Bedir M Thickness dependence of structural, electrical and optical properties of sprayed ZnO:Cu films Thin Solid Films 2008 516 1703 10.1016/j.tsf.2007.05.018

[B7] Chen M Pei ZL Sun C Gong J Huang RF Wen LS ZAO: an attractive potential substitute for ITO in flat display panels Mater Sci and Eng B 2001 85 212 10.1016/S0921-5107(01)00584-0

[B8] Gordon RG Criteria for choosing transparent conductors MRS Bull 2000 25 52 57

[B9] Nishino J Kawarada T Ohshio S Saitoh H Maruyama K Kamata K Conductive indium-doped zinc oxide films prepared by atmospheric-pressure chemical vapour deposition J Mater Sci Lett 1997 16 629 10.1023/A:1018511131738

[B10] Cooray NF Kushiya K Fujimaki A Sugiyama I Miura T Okumura D Sato M Ooshita M Yamase O Large area ZnO films optimized for graded band-gap Cu(InGa) Se2-based thin-film mini-modules Sol Energy Mater and Sol Cells 1997 49 291 10.1016/S0927-0248(97)00055-X

[B11] Kluth O Schöpe G Rech B Menner R Oertel M Orgassa K Schock HW Comparative material study on rf and dc magnetron sputtered ZnO:Al films Thin Solid Films 2006 502 311 10.1016/j.tsf.2005.07.313

[B12] Kuroyanagi A Properties of aluminum-doped ZnO thin films grown by electron beam evaporation Jpn J Appl Phys 1989 28 219 10.1143/JJAP.28.219

[B13] Groenen R Linden JL van Lierop HRM Schram DC Kuypers AD van de Sanden MCM An expanding thermal plasma for deposition of surface textured ZnO:Al with focus on thin film solar cell applications Appl Surf Sci 2001 173 40 10.1016/S0169-4332(00)00875-8

[B14] Sub ES Kang HS Kang JS Kim JH Lee SY Effect of the variation of film thickness on the structural and optical properties of ZnO thin films deposited on sapphire substrate using PLD Appl Surf Sci 2002 186 474 10.1016/S0169-4332(01)00746-2

[B15] Fu Z Lin B Zu J Photoluminescence and structure of ZnO films deposited on Si substrates by metal-organic chemical vapor deposition Thin Solid Films 2002 402 302 10.1016/S0040-6090(01)01363-3

[B16] Nunes P Fernandes B Fortunato E Vilarinho P Martins R Performances presented by zinc oxide thin films deposited by spray pyrolysis Thin Solid Films 1999 337 176 10.1016/S0040-6090(98)01394-7

[B17] Tang W Cameron DC Aluminum-doped zinc oxide transparent conductors deposited by the sol–gel process Thin Solid Films 1994 238 83 10.1016/0040-6090(94)90653-X

[B18] Zhi ZZ Liu YC Li BS Zhang XT Lu YM Shen DZ Fan XW Effects of thermal annealing on ZnO films grown by plasma enhanced chemical vapour deposition from Zn(C2H5)2 and CO2 gas mixtures J Phys D: Appl Phys 2003 36 719 10.1088/0022-3727/36/6/314

[B19] Kuo SY Chen WC Lai FI Cheng CP Kuo HC Wang SC Hsieh WF Effects of doping concentration and annealing temperature on properties of highly-oriented Al-doped ZnO films J Cryst Growth 2006 287 78 10.1016/j.jcrysgro.2005.10.047

[B20] Kim KH Park KC Ma DY Structural, electrical and optical properties of aluminum doped zinc oxide films prepared by radio frequency magnetron sputtering J Appl Phys 1997 81 7764 10.1063/1.365556

[B21] Asmar RA Juillaguet S Ramonda M Giani A Combette P Khoury A Foucaran A Fabrication and characterization of high quality undoped and Ga2O3-doped ZnO thin films by reactive electron beam co-evaporation technique J Cryst Growth 2005 275 512 10.1016/j.jcrysgro.2004.12.034

[B22] Fang GJ Li DJ Yao BL Effect of vacuum annealing on the properties of transparent conductive AZO thin films prepared by dc magnetron sputtering Phys Status Solidi A 2002 193 139 10.1002/1521-396X(200209)193:1<139::AID-PSSA139>3.0.CO;2-D

[B23] Cebulla R Wendt R Ellmer K Al-doped zinc oxide films deposited by simultaneous rf and dc excitation of a magnetron plasma: relationships between plasma parameters and structural and electrical film properties J Appl Phys 1998 83 1087 10.1063/1.366798

[B24] Hu J Gordon RG Textured aluminum-doped zinc oxide thin films from atmospheric pressure chemical‐vapor deposition J Appl Phys 1992 72 5381 10.1063/1.351977

[B25] Sanon G Rup R Mansingh A Growth and characterization of tin oxide films prepared by chemical vapour deposition Thin Solid Films 1990 190 287

[B26] Burstein E Anomalous optical absorption limit in InSb Phys Rev 1954 93 632 10.1103/PhysRev.93.632

[B27] Moss TS The interpretation of the properties of indium antimonide Proc Phys Soc Lond B 1954 67 775 10.1088/0370-1301/67/10/306

[B28] Tauc J Grigorovici R Vancu A Optical properties and electronic structure of amorphous germanium Phys Stat Sol 1966 15 627 10.1002/pssb.19660150224

[B29] Shin JH Choi DK Effect of oxygen on the optical and the electrical properties of amorphous InGaZnO thin films prepared by rf magnetron sputtering J Kor Phys Soc 2008 53 2019

[B30] Bagnall DM Chen YF Zhu Z Yao T Shen MY Goto T High temperature excitonic stimulated emission from ZnO epitaxial layers Appl Phys Lett 1998 73 1038 10.1063/1.122077

[B31] Lin B Fu Z Green luminescent center in undoped zinc oxide films deposited on silicon substrates Appl Phys Lett 2001 79 943 10.1063/1.1394173

